# Ultrafast 2D ^1^H–^1^H NMR spectroscopy of DNP-hyperpolarised substrates for the analysis of mixtures†

**DOI:** 10.1039/d1cc03079e

**Published:** 2021-07-15

**Authors:** Kawarpal Singh, Corentin Jacquemmoz, Patrick Giraudeau, Lucio Frydman, Jean-Nicolas Dumez

**Affiliations:** Department of Chemical and Biological Physics, Weizmann Institute of Science Rehovot 7610001 Israel lucio.frydman@weizmann.ac.il; Université de Nantes, CNRS, CEISAM UMR6230 Nantes F-44000 France jean-nicolas.dumez@univ-nantes.fr

## Abstract

We show that TOCSY and multiple-quantum (MQ) 2D NMR spectra can be obtained for mixtures of substrates hyperpolarised by dissolution dynamic nuclear polarisation (D-DNP). This is achieved by combining optimised transfer settings for D-DNP, with ultrafast 2D NMR experiments based on spatiotemporal encoding. TOCSY and MQ experiments are particularly well suited for mixture analysis, and this approach opens the way to significant sensitivity gains for analytical applications of NMR, such as authentication and metabolomics.

## Abstract 2

Using a fast-injection system for dissolution dynamic nuclear polarisation, ^1^H–^1^H 2D NMR spectra tailored for the analysis of mixtures are obtained in a single-scan with enhanced sensitivity.

## Graphical abstract

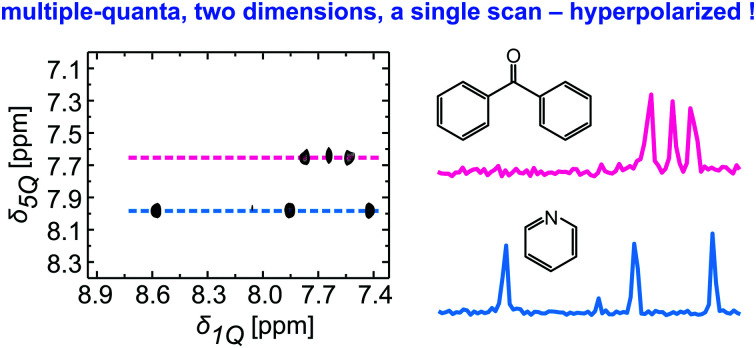

Nuclear magnetic resonance (NMR) spectroscopy is a powerful tool for the analysis of mixtures of small molecules, with applications ranging from metabolomics to forensics and quality control in pharmaceutical and food science. One of the main limitations of NMR spectroscopy is its low sensitivity, which results from the low polarisation levels of nuclear spins arising under usual experimental conditions. Several methods have been developed to prepare substrates with significantly increased polarisation of the nuclear spins. Among these, parahydrogen-based signal amplification by reversible exchange^1^ (SABRE) and dissolution dynamic nuclear polarisation^2^ (D-DNP) have already proven useful for the analysis of solution-state mixtures.^3^ However, the acquisition of 2D experiments tailored for mixture analysis, combined with broadband hyperpolarisation, remains difficult.

D-DNP is a general hyperpolarisation method originally developed, and still mainly used, in the field of biomedical imaging.^4^ In D-DNP, substrates are mixed with radicals in a glass-forming solvent, placed in a magnetic field and at liquid-helium temperature, and irradiated with microwaves.^5^ This results in a transfer of polarisation from the electron spins to the nuclear spins. The sample is then dissolved with hot solvent, and transferred to an NMR spectrometer or MRI scanner. This approach can be implemented in a robust and reproducible manner to yield signal enhancement in excess of 10 000 for heteronuclear spins such as ^13^C. However, the single-shot, *ex situ* nature of the D-DNP experiment is also associated with a number of features that limit its use in analytical applications. First, the post–dissolution decay of the spin polarisation under the effect of longitudinal relaxation, limits the kind of nuclei that can benefit from the method to species with long ∼*T*_1_s. With transfer times ranging between 2–10 s, ^1^H polarisation for many molecules will have largely decayed before the NMR data can be collected, putting a constraint on the type of molecules that can be studied. In addition, the non-renewable nature of the spin hyperpolarisation process complicates the acquisition of two-dimensional (2D) NMR data, based on the repeated acquisition of time-incremented scans: as many basic 2D experiments such as total correlation (TOCSY) and multiple-quantum (MQ) spectroscopies require full excitation, these would deplete the entire polarisation in a single scan.

In order to bypass the first of these limitations, fast-transfer methods have been developed, that reduce the time elapsed between dissolution and NMR acquisition;^6^ they also improve the latter's quality and repeatability, giving more stable injections liable to suitable pre-tuning (shimming, tuning, *etc.*). The second of the limitations can be bypassed *via* the use of ultrafast 2D NMR (UF 2D NMR) pulse sequences.^7^ UF 2D NMR relies on spatial parallelisation to collect entire 2D data set in a single scan, and this addresses the single-shot nature of D-DNP.^3*a*,*b*,*e*,8^ Its reliance on magnetic field gradients, however, makes it sensitive to turbulences and convection instabilities that can arise, in D-DNP, during and after sample injection.

In this communication we show that, with optimised fast-transfer settings, high-quality 2D ^1^H–^1^H spectra can be collected in a single scan, for mixtures of small molecules hyperpolarised with D-DNP. This is illustrated with the TOCSY experiment, that is commonly used for assignment and identification, and with 2D MQ NMR experiments, that have proven useful for the analysis of complex mixtures of aromatic compounds. Signal enhancements exceeding 100x are obtained for most peaks in these experiments. Single-scan 2D ^1^H–^1^H NMR spectroscopy of DNP-hyperpolarised substrates could thus prove useful to increase the information content of NMR experiments that target analyses of mixtures.

One of DNP's main advantages is generality: it can be used to polarise virtually any type of nuclear spins, in all kinds of chemical systems.^9^ However, the long transfer times used in most implementations imply that only long-lived signals are usually observed. Fast, pressure-driven transfer methods can broaden the range of observable nuclei and environments.^6^Fig. 1 illustrates this with conventional 1D ^1^H NMR spectra collected on quinoline, benzophenone and pyridine, as well as their corresponding thermally- and hyper-polarised 1D ^1^H NMR spectra when mixed. For performing DNP, a mixture of 3.7 M pyridine, 3 M quinoline and 1.4 M benzophenone was dissolved in DMSO-d_6_, together with the radical TEMPO at a concentration of 20 mM. A modified Hypersense polariser was used, and the sample was polarised at a magnetic field of 3.35 T at a temperature of ∼1.2 K, with microwave irradiation at a frequency of 94.150 GHz during ∼45 min. Following dissolution with hot methanol, the sample was flushed using pressurized (3.8 bar) helium. 400 μL of the post–dissolution mixture were thus pushed into a 5 mm NMR tubing waiting in the magnet bore; overall, a 3 s delay between the original dissolution and the acquisition of the first spectrum at 11.7 T was required to settle down the mixture. The use of methanol rather than D_2_O, and an optimized chase pressure and chase time, made it possible to achieve such short transfer time. The chase pressure was optimized to shorten the transfer time without compromising the settling of the sample in the NMR tube, and the chase time was chosen to inject the desired volume.

**Fig. 1:**
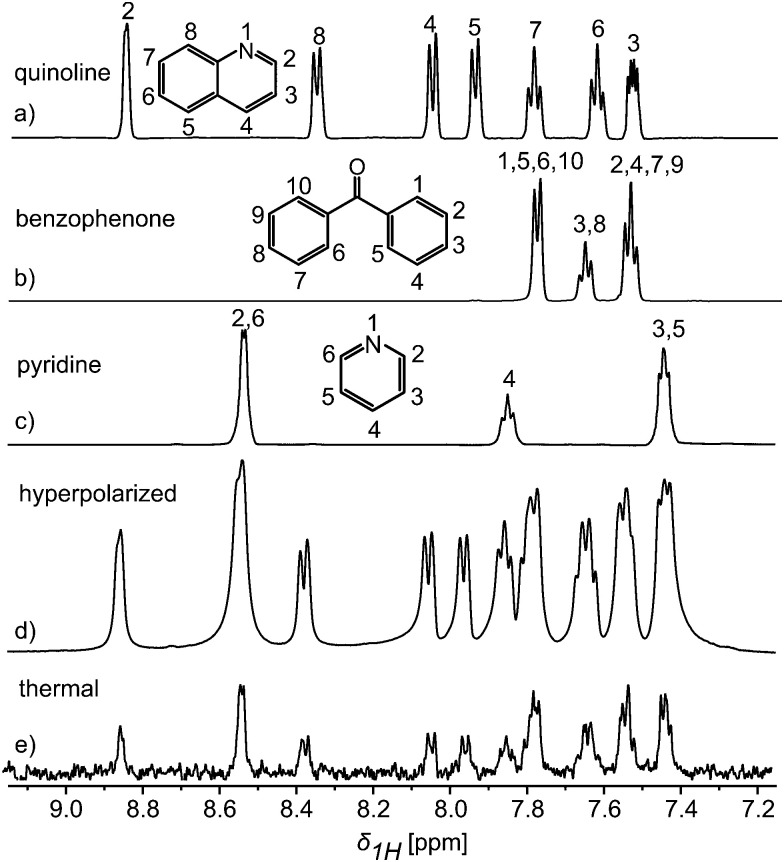
1D ^1^H NMR spectra obtained for thermally-polarized (a) quinoline (0.26 M); (b) benzophenone (0.34 M); (c) pyridine (0.38 M); (d) a hyperpolarized mixture (85 mM) of pyridine, quinoline and benzophenone at 11.7 T obtained with a small flip angle (∼1°); (e) the same sample as (d), post–dissolution, rethermalization and shimming, collected under the same acquisition conditions as (d) in a single scan. Hyperpolarization was achieved after 45 minutes of irradiation at 94.150 GHz, of a glassy sample at 1.2–1.3 K.

Fig. 1d and e compare 1D ^1^H spectra obtained from the post–dissolution mixture, before and after the loss of the hyperpolarisation. The concentrations after dissolution were in the 30–80 mM range. Both spectra were obtained with a flip angle of about 1°, and show signal enhancements of *ca.* 140–180x. The importance of a fast transfer can be appreciated by considering the timescale of the decay of the ^1^H signal. A time-series of ^1^H 1D spectra recorded after dissolution, is shown in Fig. S1 (ESI†). If the delay before acquisition were increased by 10 s, as is typical with D_2_O-based injection using standard settings,^3*d*^ the enhancement would be reduced to approximately 60. Note that the enhancement might be further increased by reducing the contribution of the radical to relaxation with, *e.g.*, a scavenger molecule, although the concentration of the radical after dissolution is already small (∼0.5 mM).^10^

The identification of components in a mixture often requires the use of 2D correlation spectra. ^1^H–^1^H TOCSY is one of the most commonly used 2D experiments for peak assignment and compound identification. The conventional, multi-scan acquisition of TOCSY spectra is not compatible with the single-shot nature of D-DNP experiments. Fig. 2a shows that high-quality TOCSY spectra can still be obtained from hyperpolarised substrates, using ultrafast 2D NMR. The corresponding pulse sequence used is shown in Fig. S2a (ESI†). A conventional 2D TOCSY spectrum collected on the same (unpolarised) sample is shown in Fig. 2b for comparison, while Fig. S3 (ESI†) shows a spectrum recorded using the same sequence and on the same mixture but after the hyperpolarisation has decayed. The DNP-enhanced UF 2D NMR spectrum was collected in a single scan lasting *ca.* 1 sec, while the conventional one was collected using 8 phase-cycled scans per *t*_1_ increment over 4 hours 26 minutes. Fig. S3 (ESI†) shows representative 1D traces extracted from these various 2D experiments along the *F*2 axis, at selected *F*1 ppm values. The different sensitivity as well as comparable quality of the acquisitions, is also evidenced from this set, with different lineshapes for UF and conventional spectra in the indirect dimension. A rigorous comparison among the data sets confirms that the correlations are suitably retrieved from the UF 2D NMR data, even if minor differences in relative cross-peak intensities between the DNP-enhanced and the thermal acquisitions arise, due to the different rates at which compounds in the mixture lose their hyperpolarisation. A signal-to-noise ratio (SNR) comparison between the two UF 2D NMR spectra shown in Fig. 2 shows that signal enhancements ≥150-fold are again obtained for all compounds after D-DNP. This is also what is observed in 1D experiments; a remarkable agreement considering the multiple gradients, and the 60 ms spin-lock period between the spatiotemporal encoding and the detection steps. It thus appears that sample convection, turbulences, or other factors that could have acted during such delay and resulted in sensitivity losses or distorted line shapes, did not act here thanks to the optimised fast transfer.

**Fig. 2:**
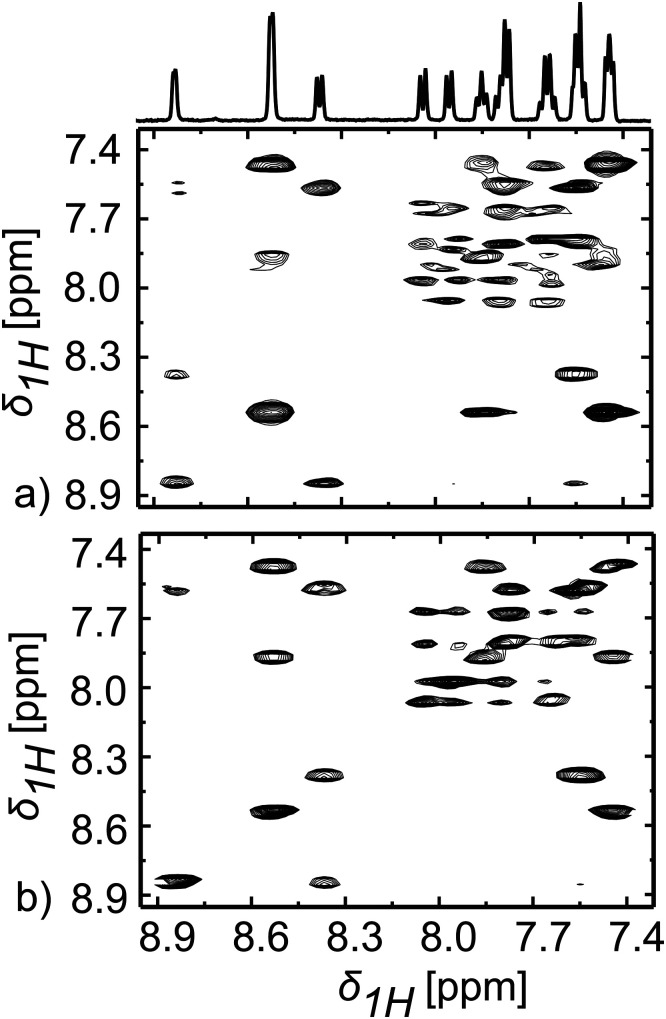
(a) 2D ^1^H–^1^H hyperpolarised UF TOCSY spectrum obtained in a single scan on the sample described in (Fig. 1d). (b) Conventional 2D ^1^H–^1^H TOCSY spectrum measured on the same sample after rethermalisation and shimming, in 4 hours and 25 minutes using 128 *t*_1_ increments with 8 scans per increment. The data for obtaining the conventional spectra were processed with same acquisition (*t*_2_) and evolution (*t*_1_) times as used for its corresponding 2D hyperpolarized UF spectra. Shown on top of the 2D sets is the conventional 1D spectrum for this mixture.

The generality of this UF ^1^H 2D approach is illustrated in Fig. 3 with the acquisition of a number of thermal and hyperpolarised 2D multiple-quantum/single-quantum (MQ/SQ) correlation spectra. These correlate along the indirect domain a *p*-quantum spectrum arising from transitions involving at least *p J*-coupled spins, with the usual ^1^H 1D spectrum of each of these spins along the direct dimension. Such experiments have been used to address complex mixtures of natural products or pollutants, as the higher-quantum spectra show increasingly simple patterns.^11^ The chemical shifts of multiple-quantum coherences can be spatiotemporally encoded.^12^Fig. 3 shows the 4Q/1Q and 5Q/1Q spectra obtained in a single scan for the hyperpolarised mixture used above, in comparison with conventional experiments (the pulse sequence is shown in Fig. S2 (ESI†), and thermal ultrafast spectra are shown in Fig. S4 and S5, ESI†). As can be seen, all the expected correlations are obtained in the DNP-enhanced UF 2D MQ NMR. Here a delay of over 100 ms had to be used between encoding and detection, for refocusing of antiphase MQ coherences into detectable, in-phase signals. Despite this, signal enhancements ranging between 110 and 190 are obtained, showing once again the good compatibility between the DNP-induced polarisation enhancement and a variety of 2D single-scan ^1^H–^1^H NMR experiments.

**Fig. 3:**
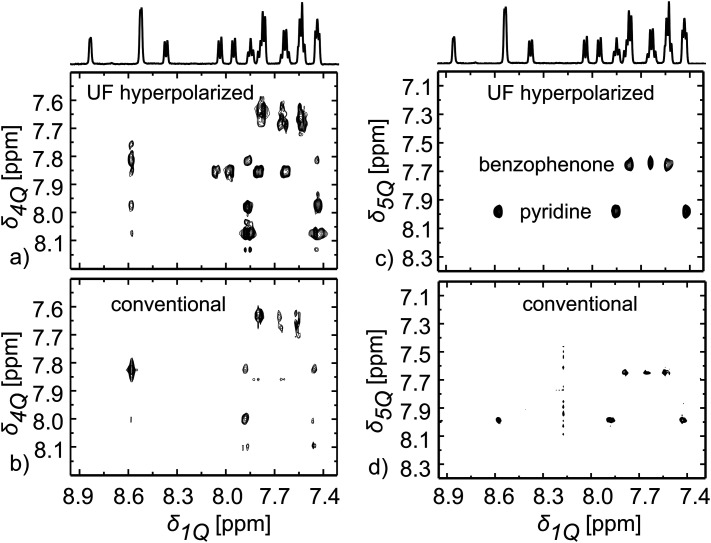
(a) 2D ^1^H–^1^H UF 4Q/1Q spectrum obtained in a single scan on a similar hyperpolarized mixture as described in Fig. 1d. (b) Conventional 2D ^1^H–^1^H 4Q/1Q spectrum measured on the same sample after rethermalisation and shimming in 8 hours and 3 minutes using 64 *t*_1_ increment steps with 32 scans per increment. (c) Same as (a) but targeting a 2D ^1^H–^1^H hyperpolarized 5Q/1Q NMR spectrum in a single scan. (d) Same as (b) but for a conventional 2D ^1^H–^1^H 5Q/1Q spectrum measured on the same sample after rethermalisation and shimming in 8 hours and 3 minutes using 64 *t*_1_ increment steps with 32 scans per increment. The data for obtaining the conventional spectra were processed with same acquisition (*t*_2_) and evolution (*t*_1_) times as used for its corresponding 2D hyperpolarized UF spectra. Shown on top of the 2D sets is the conventional 1D spectrum for this mixture.

MQ/SQ experiments become particularly useful when they are used for a “maximum-quantum” (MaxQ) analysis.^11^ The MaxQ approach relies on the fact that a system of *p* coupled spin will form a unique *p*-quantum correlation that appears as a single line in a *p*Q/1Q spectrum. Extraction of a slice from the 2D spectrum then provides the 1D ^1^H spectrum of the corresponding compound. This is illustrated in Fig. 3c, where the maxQ chemical shift for the systems of 5 spins of the benzophenone and pyridine ring can be seen. Slices taken at these chemical shifts (see Fig. S5, ESI†) provide the 1D spectra of the two compounds. In contrast, the two molecules contribute to multiple lines in the 4Q/1Q spectrum. The MQ experiment here helps to resolve overlap in the 1D spectrum (in the 1D spectrum of the mixtures, two peaks for benzophenone overlap with peaks from quinoline), and to group signals according to the spin system from which they originate.

A limitation of MQ/SQ 2D experiments is the dependence of their peak intensities on the delay chosen to generate multi-spin terms. This is illustrated here by the absence of quinoline peaks in the 5Q/1Q spectrum, in both the UF and the conventional spectrum. Optimisation methods have been described, to maximise the range of spin systems for which MQ coherences are formed.^13^ Note as well that these experiments were carried out with a probe equipped with triple-axis gradients. The use of orthogonal directions for spatial encoding and coherence-selection yields much cleaner spectra. It also makes it possible to suppress the solvent signal. As a result, undeuterated methanol could be used for dissolution for the MQ/SQ experiments. It is also interesting to note that the UF 2D NMR experiments reported here were faster than their conventional counterparts, even when accounting for the polarization time, as summarised in Table S1 (ESI†).

We have described the acquisition of high-quality 2D ^1^H–^1^H spectra, that are particularly suitable for the analysis of mixtures, for samples hyperpolarised by dissolution dynamic nuclear polarisation. The approach relies on a fast and stable sample transfer for D-DNP, that makes it possible to use single-scan 2D NMR methods based on spatial encoding, even when they involve mixing or refocusing periods of 50–100 ms. This opens the way to sensitivity gains for the vast range of analytical applications of NMR that rely on 2D experiments such as metabolomics, reaction and process monitoring, *etc.*

KS, CJ, JND: investigation, validation; CJ, KS: resources, visualisation; PG, LF, JND: funding acquisition, project administration, supervision; KS, PG, LF, JND: conceptualisation, methodology, writing.

This work has received funding from the CNRS-Israel Ministry of Science joint program (PRC CNRS no. 223851/U2NMR; IMOS-France No. 3-14888), European Research Council (ERC) under the European Union's Horizon 2020 research and innovation program (grant agreements no 801774/DINAMIX and 814747/SUMMIT), the Region Pays de la Loire (Connect Talent), the Israel Science Foundation grant 965/18, and the Perlman Family Foundation. LF holds the Bertha and Isadore Gudelsky Professorial Chair and Heads the Clore Institute for High-Field Magnetic Resonance Imaging and Spectroscopy, whose support is acknowledged. Authors from CEISAM acknowledge Estelle Martineau, the French National Infrastructure for Metabolomics and Fluxomics MetaboHUB-ANR-11-INBS-0010 (www.metabohub.fr) and the Corsaire metabolomics core facility (Biogenouest).

## Conflicts of interest

There are no conflicts to declare.

## Supplementary Material

CC-057-D1CC03079E-s001
