# Supplementary material for: Ultrafast 2D 1H–1H NMR spectroscopy of DNP-hyperpolarised substrates for the analysis of mixtures
Source: Chem Commun (Camb). 2021 Jul 15;57(65):8035–8. doi: 10.1039/d1cc03079e (PMC8477446; doi:10.1039/d1cc03079e)
Supplement: CC-057-D1CC03079E-s001 [file CC-057-D1CC03079E-s001.pdf]

Electronic Supplementary Material (ESI) for ChemComm.
This journal is © The Royal Society of Chemistry 2021

Supplementary Information for "Single-scan 2D ^{1}H-^{1}H NMR spectroscopy of DNP-hyperpolarised substrates for the analysis of mixtures"

Kawarpal Singh,[a] Corentin Jacquemmoz,[b] Patrick Giraudeau,[b] Lucio Frydman[a,*] and Jean-Nicolas Dumez[b,*]

[a] *Department of Chemical and Biological Physics, Weizmann Institute of Science, 7610001, Rehovot, Israel*

[b] *Université de Nantes, CNRS, CEISAM UMR6230, F-44000 Nantes, France*

Contents

Materials and methods 2

Supplementary figures 4

Supplementary table 9

Materials and methods

Sample preparation: All the chemicals and solvents were purchased from Sigma-Aldrich and used as received. Mixtures of 3.7 M pyridine, 3 M quinoline, 1.4 M benzophenone and 0.2 mL of DMSO-d_6 with 20 mM TEMPO (2,2,6,6-Tetramethylpiperidin-1-yl) radical were polarized in a HyperSense polarizer (3.35 T, Oxford Instruments Molecular Bio tools, UK) as described below. After being vortexed, the 120 µL DNP sample was sonicated for 5 minutes before insertion into the polarizer**.**

Dynamic Nuclear Polarization: The samples were hyperpolarized using an Oxford Instruments Hypersense. An additional EH-500 Edwards booster pump was used along with Oxford Supplied E2M80 vacuum pump. This combination lowers the operating pressure to ~1 mbar. The temperature achieved for polarization was ∼1.1−1.26 K with the booster on, vs ∼1.4 K−1.60 K without the booster. ^{1}H DNP was achieved by irradiating microwaves to the sample at 94.150 GHz for ~45 min at power levels of 180 mW. Following irradiation, the samples were dissolved using 4 mL of solvent pressurized to 9 bar and temperature to approximately 160 °C. Undeuterated methanol was used for hyperpolarized 2D UF MQ-SQ experiments and methanol-d_4 for the 2D UFTOCSY experiments. The sample was transferred to the NMR tube by the helium chase gas with 3.8 bar pressure.

NMR Spectroscopy: A triple-axis gradient “HCN” probe was used in a 11.7 T Magnex magnet interfaced to a Varian iNova console. The probe is capable of generating z gradients up to 30 G/cm and x, y gradients up to 60 G/cm. Both the 1D and 2D NMR experiments were triggered upon injecting the hyperpolarized sample in the 5 mm tube waiting inside the magnet bore. The signal enhancement upon polarization was obtained using series of 1D ^{1}H NMR spectra collected using small flip angle excitation (∼1°). The desired peak intensities were integrated and normalized with the thermal equilibrium values to obtain the signal enhancement vs. time data. Also, a hyperpolarized 1D ^{1}H NMR spectrum was obtained 0.5 seconds before the acquisition of each hyperpolarized 2D experiment to validate the enhancement and stability of the injected sample, using a small flip angle of ∼1°.

Ultrafast MQ-SQ 2D correlations: For the UF MQ-SQ experiments, encoding gradients of amplitude 0.65 G/cm and 0.28 G/cm were used for obtaining 4Q and 5Q spectrum respectively. A chirp pulse of sweep width of 12 kHz with duration $T_e/2$ of 15 ms was used. A pair of orthogonal gradients of length 600 µs and amplitude 4 G/cm was placed around the second chirp pulse for both experiments. Coherence selection gradients of amplitude 5 G/cm and length 5 ms were used along the y axis for 4Q and of amplitude 5 G/cm and length 8 ms were used for 5Q experiments. The two experiments followed the $G_2 = p \times G_1$ rule where p is the coherence order selected in the experiment. An acquisition gradient (G_a) of amplitude 8.69 G/cm and duration 432 µs was used for 4Q while 10.32 G/cm with duration 396 µs was used for 5Q. The number of loops for 5Q and 4Q experiments was 123 and 160 respectively. The acquisition time for 5Q and 4Q was 97 ms and 138 ms respectively. A multiple quantum build-up time (2τ) of 166.4 ms, a refocusing delay of 145 ms, and a purge gradient (G_p) of 482 µs with amplitude of 18 G/cm were used for the 4Q experiment. Similarly, a multiple quantum build-up time (2τ) of 203.94 ms, a refocusing delay of 120 ms, and a purge gradient of 396 µs with amplitude of 13 G/cm were used for the 5Q experiment. The build-up and refocusing times were determined using the procedure used by Concillio et. al. (ChemPhysChem 2018, 19, 3310). The hyperpolarized UF MQ-SQ experiments were done in a single scan in less than a second and thermal experiments were done after the hyperpolarized experiment using the same sample by shimming using 512 scans for 4Q in 2 hours and 29 minutes and 5Q spectrum was obtained with 3398 scans in 9 hours and 56 minutes.

Ultrafast TOCSY experiment: An encoding gradient (G_e) of amplitude 2 G/cm with chirp pulse of sweep width 15 kHz and duration $T_e/2$ of 20 ms were used. An acquisition gradient (G_a) of 14 G/cm with duration of 340 µs and 123 loops was used, leading to an acquisition time of 83.64 ms. A dipsi2 mixing pulse was applied for 60 ms. The thermal ultrafast TOCSY experiment for the post dissolution sample was done with 256 scans.

Conventional MQ-SQ: Both conventional 2D 4Q and 5Q spectra were obtained with 64 t_1 increment steps with 32 scans per increment and repetition time of 10 seconds in 8 hours and 3 minutes.

Conventional TOCSY: It was recorded with States-Haberkorn-Ruben method with a repetition time of 7 seconds using 128 t_1 increments with 8 scans per increment. The experiment was done in 4 hours and 25 minutes.

Data processing: Multiple quantum chemical shifts were represented in a reduced chemical shift scale for the indirect dimension by dividing the chemical-shift axis in the indirect dimension by the coherence order of that MQ experiment. The evolution time of 30 ms was set for both UF and conventional experiments with acquisition time of 97 ms for 5Q and 138 ms for 4Q. The multiple quantum build-up times were the same for conventional and ultrafast experiments. The 2D ultrafast TOCSY and its conventional counterpart were processed by setting the evolution time of 40 ms and acquisition time of 83 ms. Gaussian apodization was used in the ultrafast dimension while exponential apodization was used in the conventional dimension.

Supplementary figures

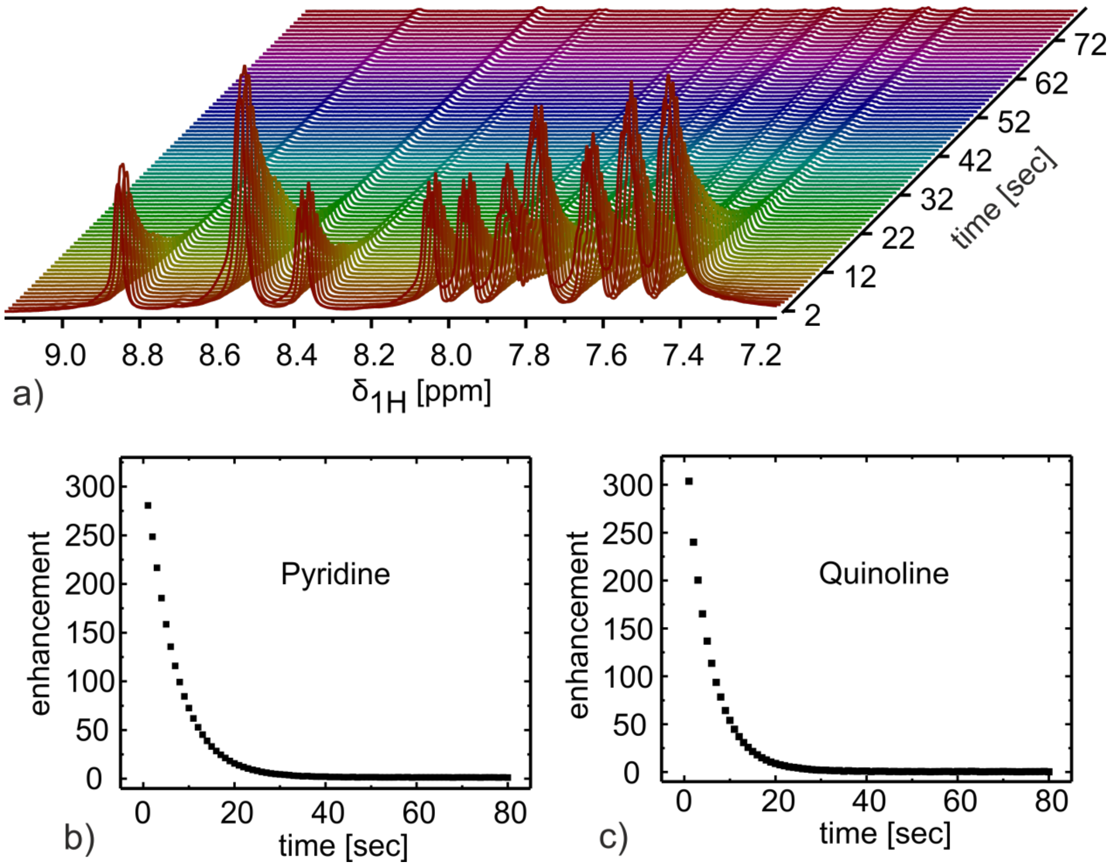

Figure S1. a) Stacked plot obtained by acquiring series of 1D ^{1}H spectra acquired with small flip angle (~ 1°) of excitation. The hyperpolarized sample was injected into the 11.7 T after the dissolution and polarization in the Hypersense polarizer. Signal enhancement obtained by integrating and normalizing the desired peak of b) Pyridine, and c) Quinoline.

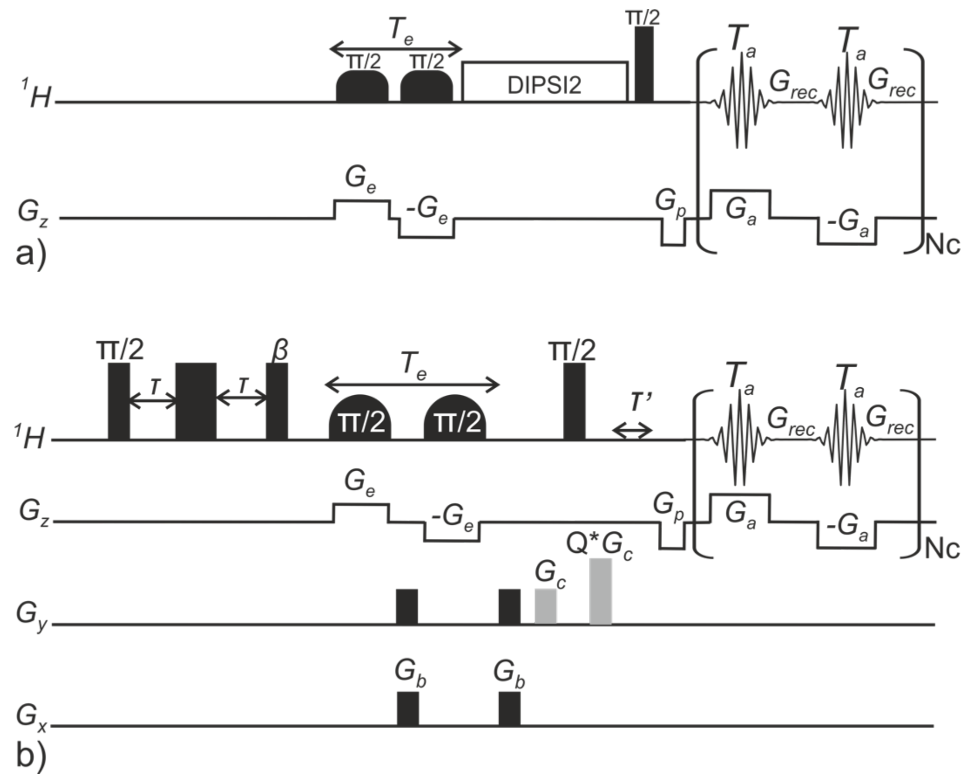

Figure S2 a) Pulse sequence used in the 2D UF TOCSY ^{1}H NMR acquisition. b) Pulse sequence used in the 2D UF MQ/SQ ^{1}H NMR acquisitions; shaded gradients were calibrated to select the desired coherence order.

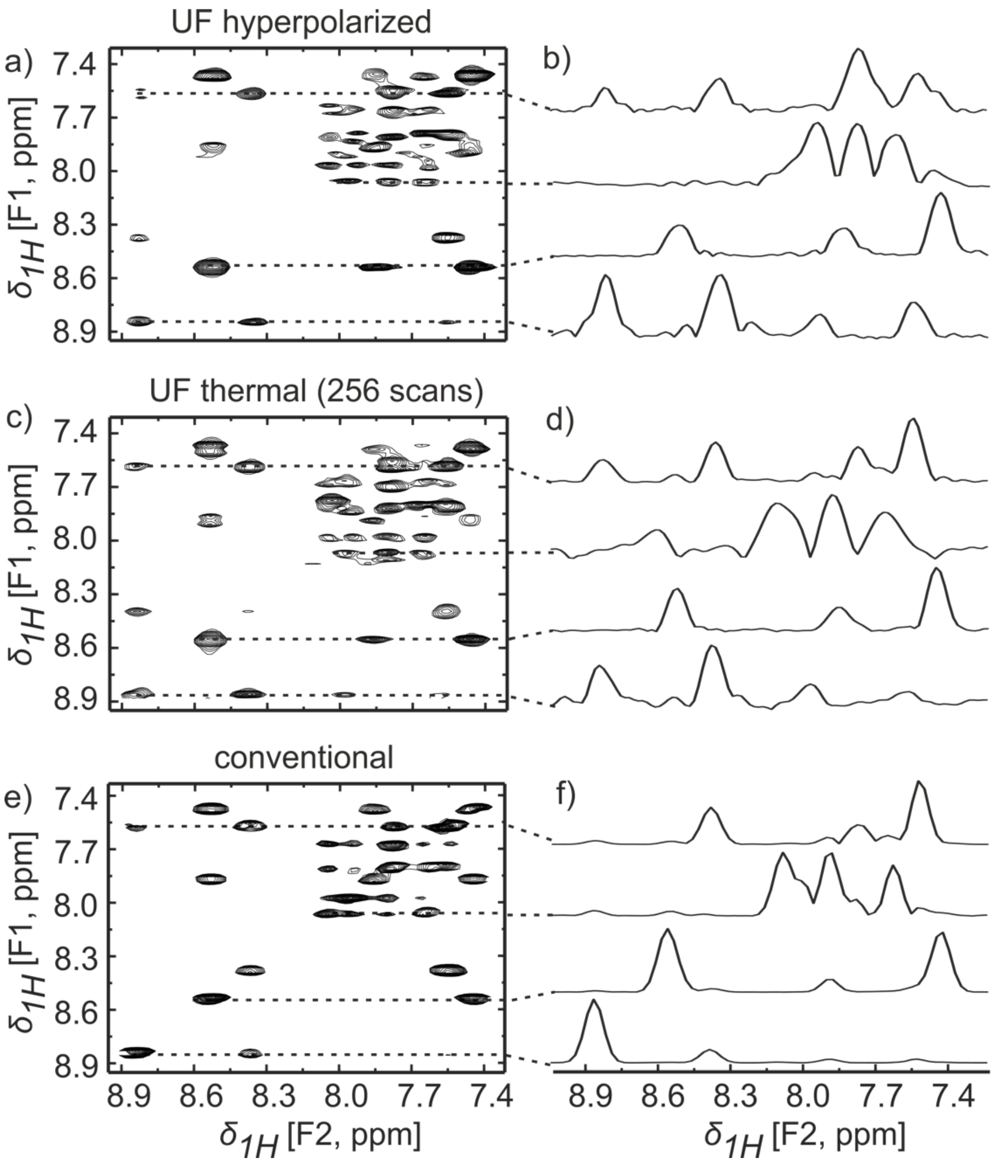

Figure S3: a) a) Hyperpolarised 2D ^{1}H-^{1}H TOCSY spectrum obtained after the polarization and injection into the 11.7 T magnet and b) 1D traces extracted corresponding to chemical shifts in the F1 dimension. c) Thermal 2D ^{1}H-^{1}H TOCSY spectrum measured on the same sample after rethermalisation and shimming and d) 1D traces corresponds to chemical shift in the F1 dimension.[a] e) Conventional 2D ^{1}H-^{1}H TOCSY spectrum measured on the same sample as in thermal experiment using 64 t_1 increment steps with 32 scans per increment and f) 1D traces corresponds to chemical shift in the F1 dimension.

[a] Note that the broader lineshape for the second slice from the top is due to a k=0 artefact in the thermal UF spectrum.

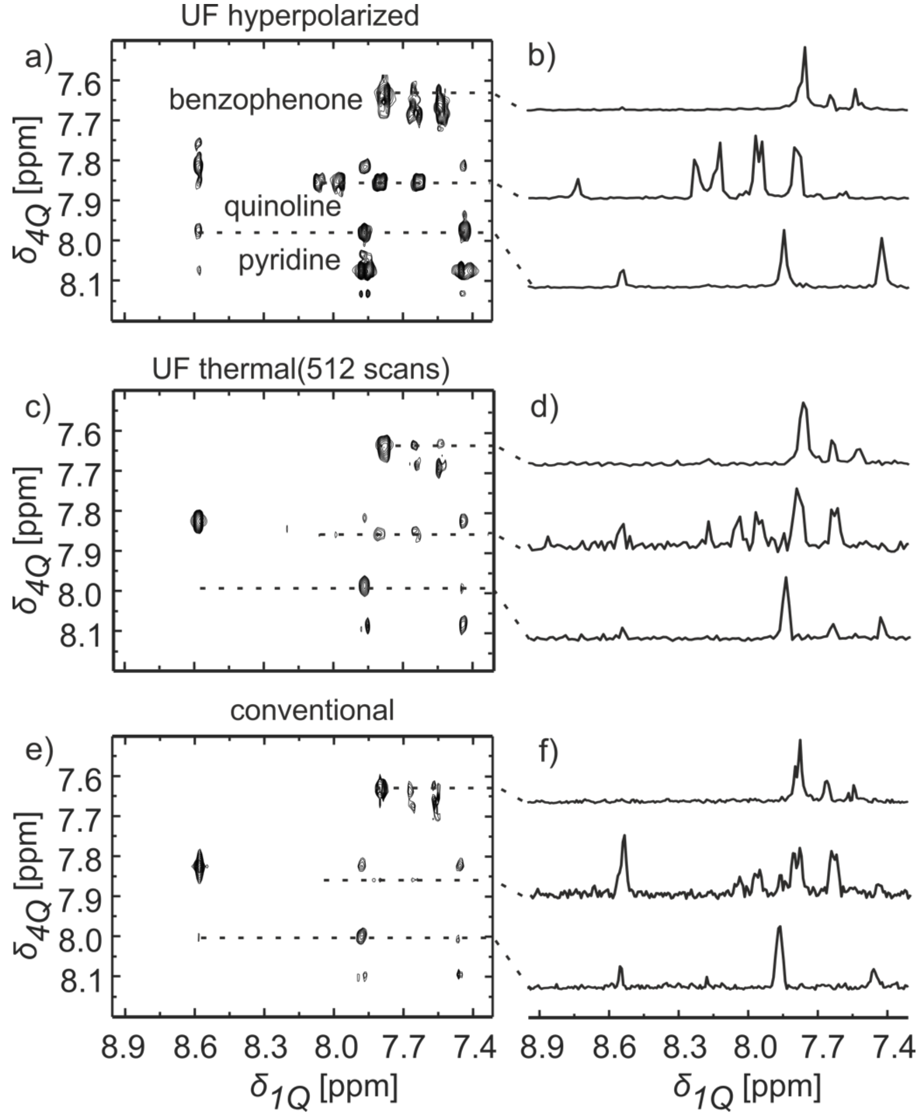

Figure S4 a) Hyperpolarised 2D UF ^{1}H-^{1}H 4Q spectrum obtained after the polarization and injection into the 11.7 T magnet and b) 1D traces extracted corresponding to 4Q chemical shifts in the F1 dimension. c) Thermal 2D ^{1}H-^{1}H UF 4Q spectrum measured on the same sample after rethermalisation and shimming and d) 1D traces corresponds to 4Q chemical shift. e) Conventional 2D ^{1}H-^{1}H 4Q spectrum measured on the same sample as in c) using 64 t_1 increment steps with 32 scans per increment and f) 1D traces corresponds to 4Q chemical shift.

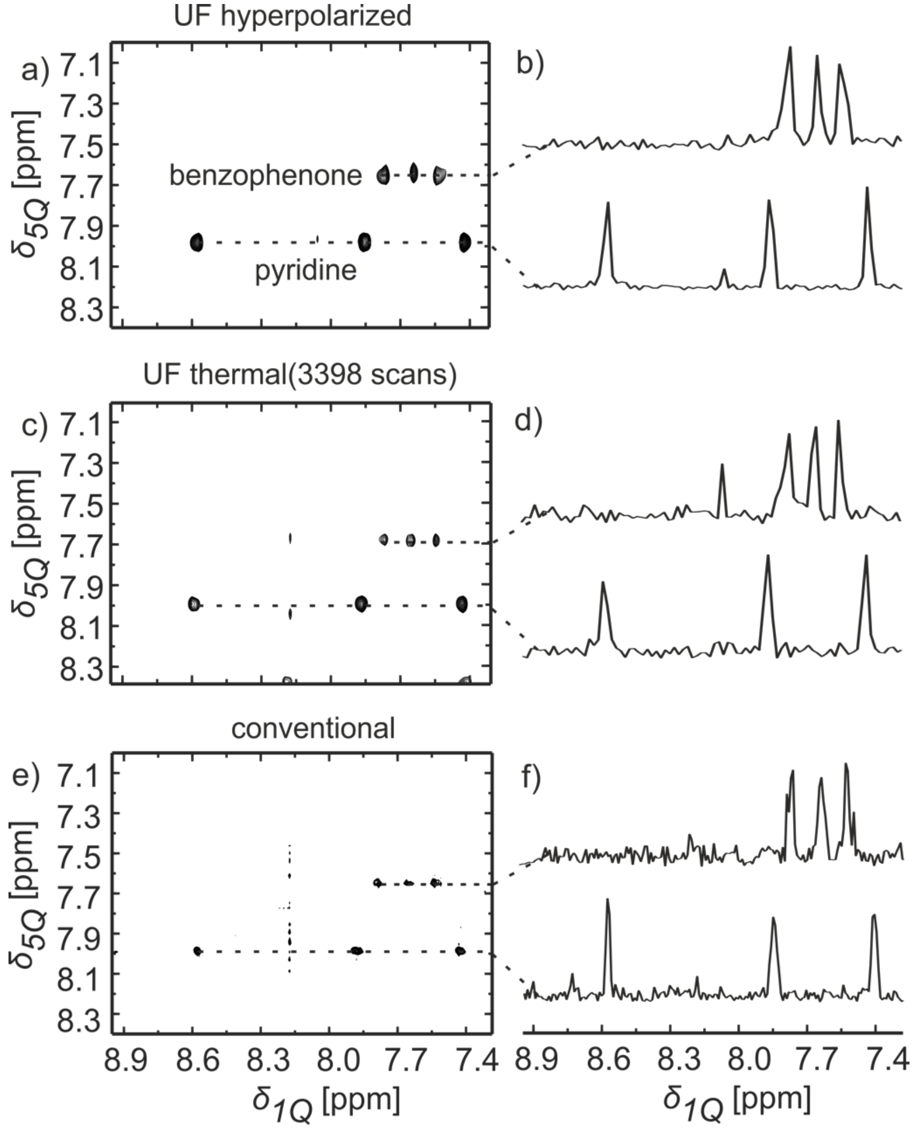

Figure S5 a) Hyperpolarised 2D UF ^1H-^1H 5Q spectrum obtained after the polarization and injection into the 11.7 T magnet and b) 1D traces extracted corresponding to 5Q chemical shifts in the F1 dimension. c) Thermal 2D ^1H-^1H UF 5Q spectrum measured on the same sample after rethermalisation and shimming and d) 1D traces corresponds to 5Q chemical shift. e) Conventional 2D ^1H-^1H 5Q spectrum measured on the same sample as in c) using 64 t_1 increment steps with 32 scans per increment and f) 1D traces corresponds to 5Q chemical shift.

Supplementary table

Table S1 Total duration of the experiments, including, in the hyperpolarised case, the polarisation time. The numer of scans and increments for the UF thermal and conventional experiments are given in the Materials and methods section above.

	**UF hyperpolarized**	**UF thermal**	**Conventional**
2D ^{1}H-^{1}H TOCSY	45 min	50 min	4 h 25 min
2D ^{1}H-^{1}H 4Q/1Q	45 min	2 h 29 min	8 h 3 min
2D ^{1}H-^{1}H 5Q/1Q	45 min	9 h 56 min	8h 3 min
